# Low-coherence photonic method of electrochemical processes monitoring

**DOI:** 10.1038/s41598-021-91883-z

**Published:** 2021-06-15

**Authors:** Monika Kosowska, Paweł Jakóbczyk, Michał Rycewicz, Alex Vitkin, Małgorzata Szczerska

**Affiliations:** 1 Department of Metrology and Optoelectronics, Faculty of Electronics, Telecommunications and Informatics, Gdańsk University of Technology, 11/12 Narutowicza St, 80-233 Gdańsk, Poland; 2 Department of Medical Biophysics, University of Toronto, 101 College St, Toronto, ON M5G 1L7 Canada; 3 Princess Margaret Cancer Centre, University Health Network, 610 University Ave, Toronto, ON M5G 2C1 Canada; 4 Department of Radiation Oncology, University of Toronto, Stewart building, 149 College St Suite 504, Toronto, ON M5T 1P5 Canada

**Keywords:** Optoelectronic devices and components, Optical sensors, Electrical and electronic engineering

## Abstract

We present an advanced multimodality characterization platform for simultaneous optical and electrochemical measurements of ferrocyanides. Specifically, we combined a fiber-optic Fabry–Perot interferometer with a three-electrode electrochemical setup to demonstrate a proof-of-principle of this hybrid characterization approach, and obtained feasibility data in its monitoring of electrochemical reactions in a boron-doped diamond film deposited on a silica substrate. The film plays the dual role of being the working electrode in the electrochemical reaction, as well as affording the reflectivity to enable the optical interferometry measurements. Optical responses during the redox reactions of the electrochemical process are presented. This work proves that simultaneous opto-electrochemical measurements of liquids are possible.

## Introduction

Global research trends are aimed at combining multiple measurement techniques using different modalities. This increases the attractive possibility of obtaining more information because of the synergy between different methods. More and complementary data can be correlated to yield new insights into the investigated samples or processes^1,2^. For example, the combination of electrochemical and optical methods appears very promising, as these are characterized by high sensitivity and accuracy as well as the possibility of miniaturization and automation.

Different approaches to combining these methods have been proposed in the literature. Lamberti et al. presented an optoelectrochemical sensor applying modified graphene^3^, fluorescence and electrochemical impedance spectroscopies were combined to monitor insulin levels. Narakathu et al. built a system with a dual detection of heavy metals^4^; the electrochemical measurement was performed with electrochemical impedance spectroscopy with the use of gold interdigitated electrodes, and the optical readout was based on Raman spectroscopy. Sobaszek et al. described a setup for in situ monitoring of electropolymerization processes occurring at boron-doped diamond electrode with the use of on optical Mach–Zehnder interferometer^5^; the authors report a spectral shift resulting from surface electrode modification with melamine. Janczuk-Richet et al. constructed a setup for electrochemical reactions investigations using fiber Bragg gratings coated with ITO (indium tin oxide)^6,7^; ITO plays the role of a working electrode in an electrochemical system and the coating increases the sensitivity of Bragg gratings to refractive index changes in the optical subsystem. Liu et. al presented a setup joining total internal reflection ellipsometry with an electrochemical setup^8^. This solution allows for monitoring of sub-surface changes during electrochemical reactions. Cobet et al. proposed ellipsometric spectroelectrochemical system for in situ investigation of polymer doping^9^. Our research assessed the sample state during the electrochemical cycle where any perturbations in the investigated liquid’s refractive index indicate changes due to the EC reactions. In our study, we monitor the liquid sample itself, not the electrode surface. We have shown that the hybrid diagnostic system allows for a great reduction of the required sample volume. The quick operation, robustness, low cost and simple construction additionally distinguish our methodology from others.

Electrochemistry (EC) is of great interest in such systems as it is an extremely useful approach in technology^10–12^. Its important usages include the detection of organic compounds and chemicals down to very small concentrations (depending on materials used for the system construction and the targeted substance, detection limit of 1 µg L^−1^ or lower is achievable^13,14^), the treatment of electrode surfaces, and degradation of dangerous substances. Among electrochemical techniques, voltammetric methods can be distinguished. They are based on the investigation of the current dependence on applied voltage^15^. Changing voltage causes molecules to chemically gain or lose electrons, meaning they can undergo oxidation and reduction processes. As a result, current flow is registered as a function of applied voltage creating an electrochemical curve (voltammogram). Voltammetry methods are characterized by high sensitivity and specificity towards molecules^16^. Portable and relatively cheap apparatus can be used for the system construction, and a large variety of electrodes can be applied to best match the desired application.

Considering now the optical characterization methods, fiber-optic sensors are characterized by small dimensions allowing their applications in difficult-to-access places. They are also immune to electromagnetic interferences. Non-contact measurements and transmission of the data over long distances are possible. Further, analysis of signals in the spectral domain, such methods are largely insensitive to changes in optical transmission (signal intensity fluctuations) occurring in the measurement path. Owing to these and many other advantages, fiber-optic sensors are widely used in science and industry, including biological, chemical and environmental applications^17–22^. One of the methods that benefit from fiber-optic implementation is low-coherence interferometry. This method ensures high measurement dynamic and resolution while eliminating the problem of ambiguous results as absolute measurement values are provided^23,24^. For instance, optical fibers were used to construct a tandem-type interferometer utilizing a low-coherence method to monitor a metalorganic vapor-phase epitaxy process: substrate bending and temperature were monitored as well as the layer thickness^25^. A low-coherence interferometry was also applied to monitor growth kinetics of diamond growth during microwave plasma chemical vapor deposition process where thickness and growth rate were controlled^26^.

Our previous research on fiber-optic sensors presented a measurement module employing boron-doped diamond (BDD) film as a mirror^27,28^. The results demonstrate that optical spectra are modulated by changes in the refractive index of the investigated liquid inside the cavity bounded by the BDD film, and by the change in the cavity length comprised of the investigated liquid. Our finding that the BDD film can successfully serve as a reflective layer in the interferometer opened a way to construct a hybrid opto-electrochemical system. The combination of these two methods has great potential, as BDDs are widely used as working electrodes in electrochemical cells^29^. They are popular because such electrodes are characterized by a high response reproducibility, chemical inertness, wide electrochemical potential window—broader than graphite and glassy carbon^30^, low and stable capacitive background current, long-time response stability, and biocompatibility^31^. BDD can be found in electrodes used in biosensors for the detection of various compounds such as glucose, antibiotics, amines, proteins and DNA^32–35^. Further, their application is not only limited to detection; they are also successfully used for the degradation of dangerous chemical compounds^36,37^ and wastewater treatment^38^. Extraordinary properties of BDD enable its performance as a working electrode and a mirror, making it unique and crucial for integration of a Fabry–Perot interferometer with an electrochemical system.

In this paper, we present a hybrid setup for simultaneous measurements of liquid samples by interferometric and electrochemical methods. We show that such construction is possible thanks to the crucial part that links up these two techniques: a boron-doped diamond film. This material has extraordinary electrochemical and optical properties allowing its usage as a joining element playing a dual role of a working electrode and a reflective surface. The system is comprised of a Fabry–Perot fiber-optic interferometer and a potentiostat–galvanostat with a 3-electrode electrochemical cell. This approach permits the monitoring of the sample state during the electrochemical cycle by means of optical interferometry and may potentiate real-time feedback. Such an approach has not been reported before, to the best of the authors’ knowledge, and will enable relatively cheap and simple configuration with the possibility of miniaturization.

## Results

**Figure 1:**
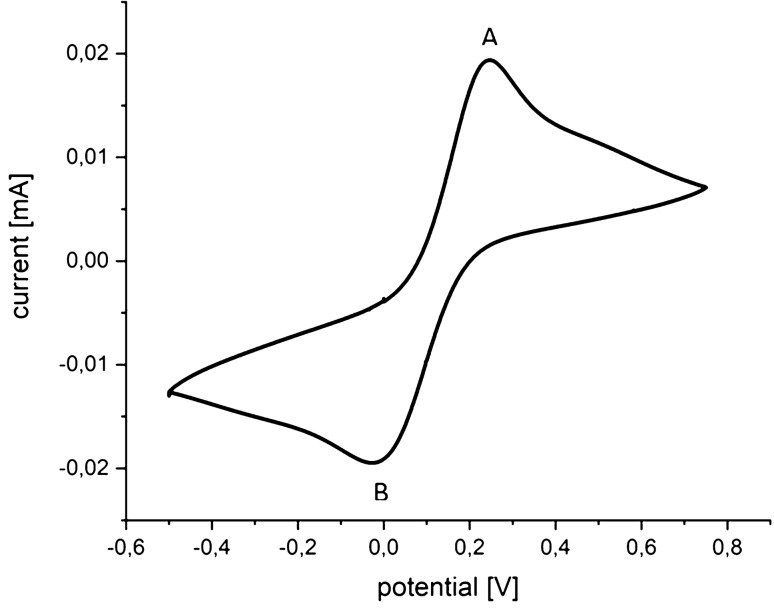
Representative cyclic voltammetry response recorded with a BDD film as a working electrode. Redox reactions were carried out using K_3_[Fe(CN)_6_] in a Na_2_SO_4_ solution. Expected oxidation and reduction peaks are seen (points A and B, respectively), suggesting proper EC functionality of the hybrid EC-optical system (see text for details).

We performed optical and electrochemical measurements during redox reactions by means of cyclic voltammetry using 2.5 mM K_3_[Fe(CN)_6_] in a 0.5 M Na_2_SO_4_ solution. During the electrochemical measurements, optical spectra were recorded simultaneously. The representative electrochemical response is shown in Fig. 1. The resulting curve is consistent with standard EC behavior^15,39^, suggesting the proper operation of the constructed system. For example, the oxidation (point A: E = 0.247 V, I = 0.0195 mA) and reduction peaks (point B: E = − 0.027 V, I = − 0.0194 mA) peaks, for a scan rate of 10 mV/s, correspond to the presence of Fe(CN)_6_^3−^ and Fe(CN)_6_^4−^ ions, respectively. Such visible peaks for oxidation (A—peak anodic current) and reduction (B—peak cathodic current) are typical for a reaction that is rate-dependent on the diffusion of the analyte to and from the planar working electrode surface^39^. Achieving the correct current–voltage voltammetry response in presence of the optical system suggests that the two sub-systems do not interfere with each other, and can be used successfully for opto-electrochemical measurements.

**Figure 2:**
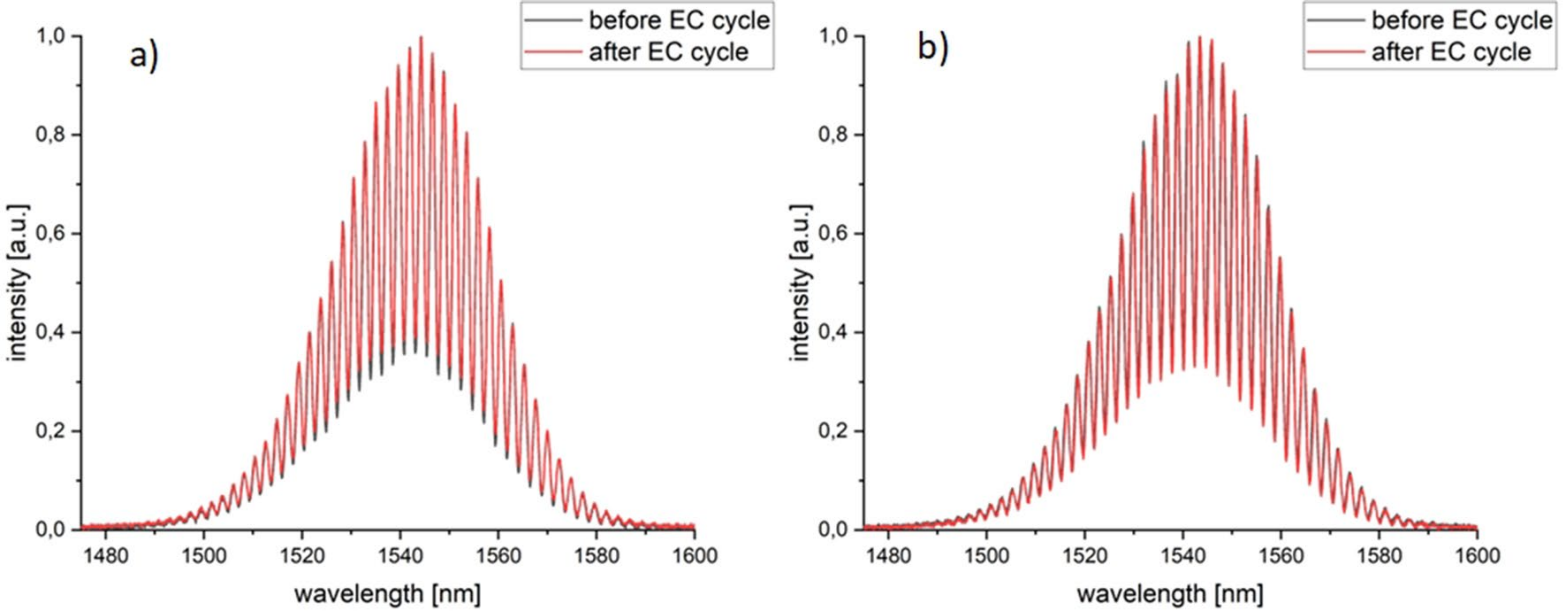
Optical spectra obtained before and after full electrochemical cycle. Spectra (**a**) and (**b**) show two out of five consecutive voltammetric cycles. Note the excellent overlap for the pairs, and the similarity of spectal pairs to each other across the longitudinal measurements, indicating that no significant perturbation appeared during measurements and the investigated sample (2.5 mM K_3_[Fe(CN)_6_] in a 0.5 M Na_2_SO_4_) was not damaged. The results indicate the successful performance of the BDD film in its dual role of a EC working electrode and an optically reflective layer.

Simultaneously with the cyclic voltammetry measurements, optical spectra were recorded, before and after the electrochemical cycle. The procedure was repeated five times. The measurements were taken with the use of a 1550 nm light source and recorded with an optical spectrum analyser. The resultant spectra are shown in Fig. 2.

With the Fabry–Perot interferometer we can detect changes of absorption and refractive index of the investigated liquid as a result of the electrochemical reaction. Potential adsorption of a newly formed chemical compound to a BDD will also influence the optical spectra. It can be noted that optical responses registered before and after the full electrochemical cycle are overlapping as expected; this holds true for every repeated measurement pair for the ~ 1-min duration of the experiment. This means that no significant change in the investigated sample occurred—if the refractive index of the liquid would change during the process, the spectra will be modified. Visible change of the spectra will also appear if reaction products are adsorbed onto the BDD film surface. The constancy of the optical response before and after EC and across repeated measuremetns implies that the sample was not damaged and optically confirms the reversibility of the electrochemical process.

**Figure 3:**
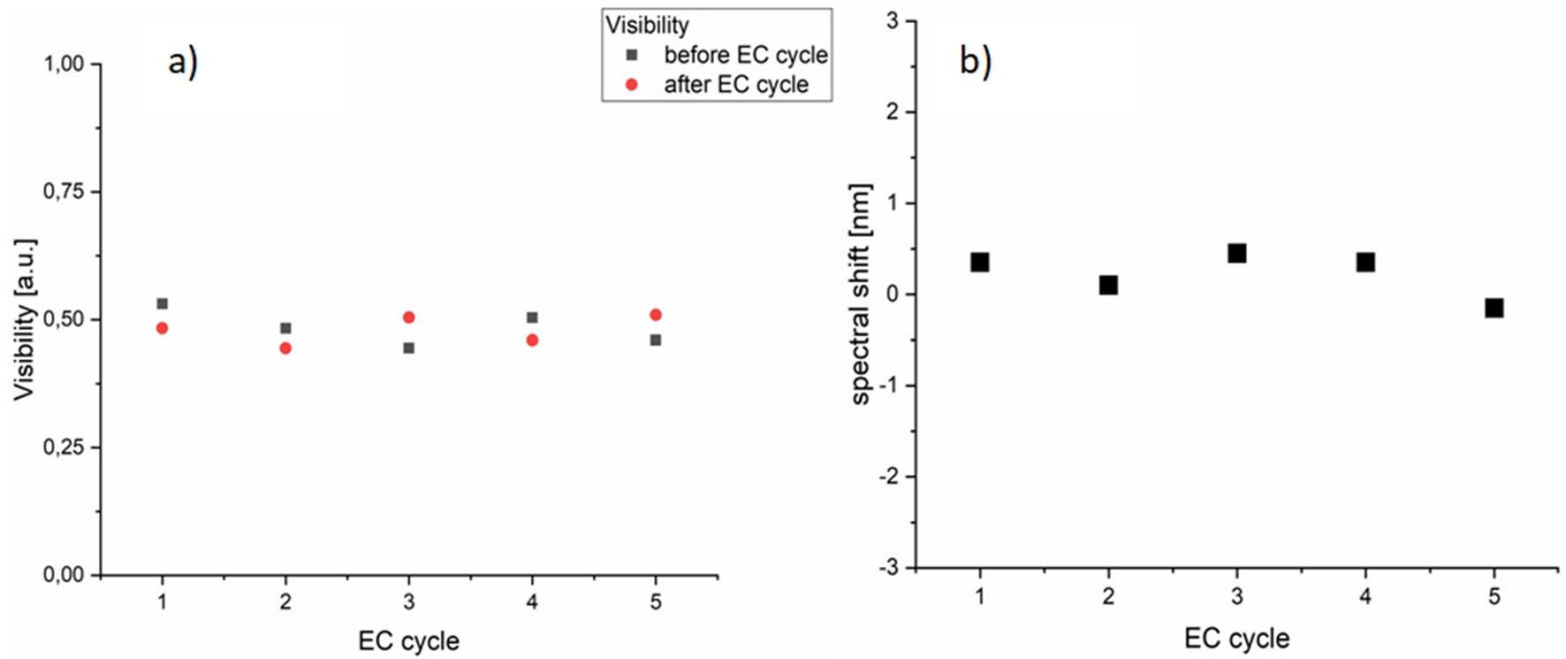
The analysis of the optical spectra of Fig. 2 recorded before and after each electrochemical cycle. (**a**) Fringe visibility values cluster around ~ 0.5 value due to the presence of the liquid inside the Fabry–Perot cavity. (**b**) Spectral shift between spectra recorded before and after each EC cycle. Minor changes in (**a**) and (**b**) are due to instability of the light source over time.

To quantify this constancy further, the signal fringe visibility as well as spectral shift between spectra recorded before and after the EC cycle were calculated and are shown in Fig. 3. In interferometry, fringe visibility V describes the contrast of the obtained signal, and can be calculated using the maximal I_max_ and minimal I_min_ intensity values as follows: V = (I_max_ − I_min_)/(I_max_ + I_min_). For the optimal cavity length in air, values approaching unity can be obtained for the BDD film^27^. In our experiment, with the cavity containing 2.5 mM K_3_[Fe(CN)_6_] in a 0.5 M Na_2_SO_4_ solution, a median value of V = 0.48 was obtained. This is due to the presence of the liquid inside the cavity and resulting absorption loss lowering the visibility value. From Fig. 3b. it can be noted that a small (< 0.5 nm) spectral shift occurs, likely stemming from the small instability of the light source.

**Figure 4:**
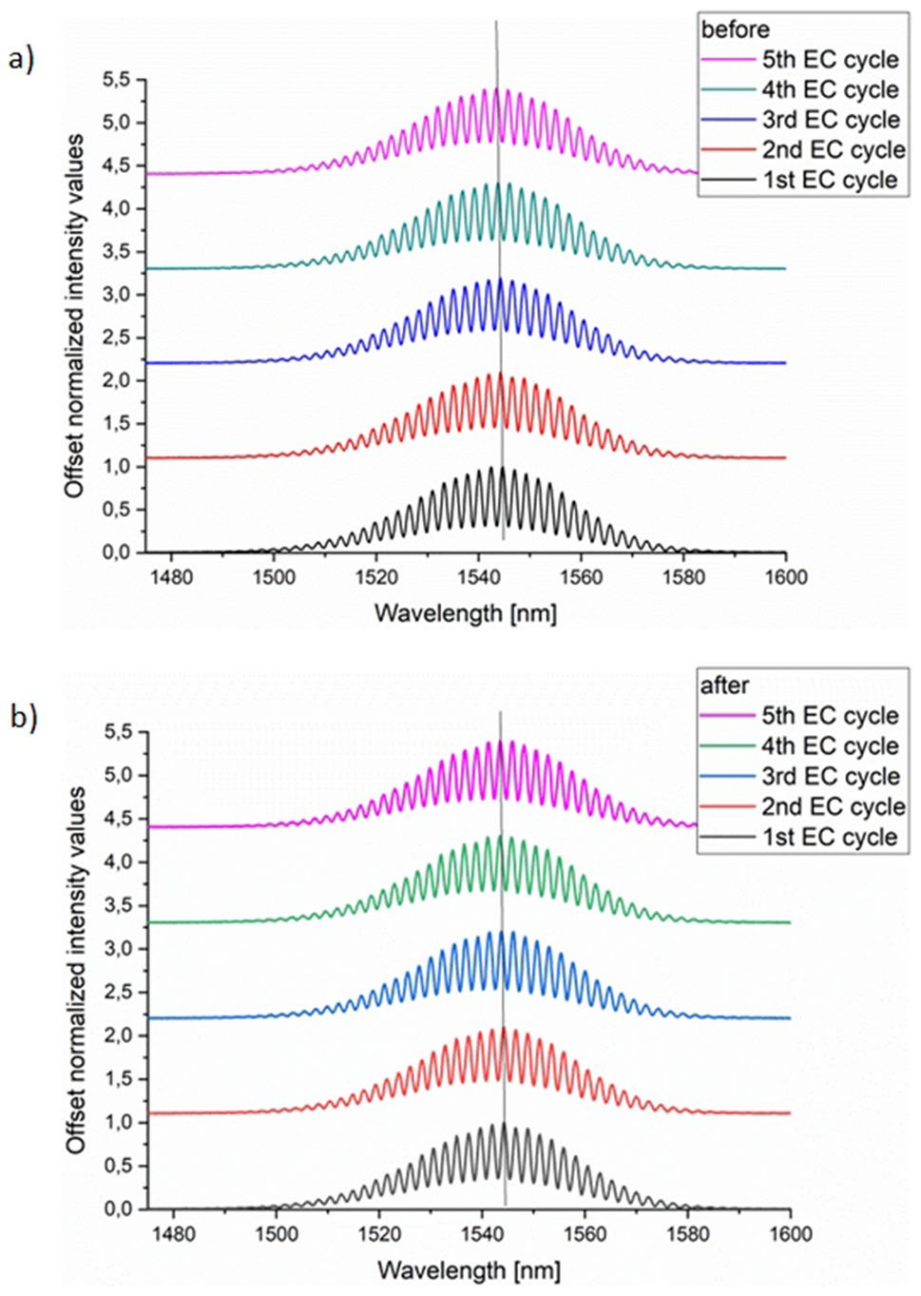
Stacked spectra split into *before* and *after* according EC cycles groups. The results allow for comparison of signals obtained in the time series of repeated measurements. As seen, the signals’ envelopes remain approximately the same, suggesting no measurement-induced alterations were introduced to the electrochemical reactions. Vertical lines are to guide an eye to spot slight spectral shifts, if any (likely due to the light source instability).

Figure 4 presents all spectra obtained for five repeated-measurement electrochemical cycles, divided into two groups: starting spectra registered before each EC cycle, and ending spectra registered after completing each EC cycle. As seen, no additional modulation occurs in optical signals for later measurements compred to the earlier ones, and the signal envelopes remain the same. These results confirm the repeatability of the measurements in the constructed opto-electrochemical system during the reversible redox reactions.

## Discussion

We have shown that it is possible to perform optical *and* electrochemical measurements in the proposed hybrid system. The obtained optical spectra remain stable during the measurements, implying that the electrochemical redox processes were carried out without any hysteresis or other irreversible altrations. Indeed, if the refractive index change of the investigated liquid was induced by the hybrid measurement process, the spectra would be altered. As the reversible process is presented, no visible spectrum modulation was observed, at least within the resolving power of our measurement system, we conclude that our hybrid system indeed operates in a true non-destructive evaluation (NDE) mode. This ensures us that the entire process was successful and that the sample was not damaged, which saves resources: sample (small volumes) and time (rapid monitoring and feedback). We can further optimize the process while maintaining low cost. For reversible reactions, we achieve the lowest measurements cost and we can optimize them as liquid and the working electrode can be used many times, since the substance quality is continuously monitored. For irreversible EC processes, we cannot use the same sample multiple times: the liquid properties change and the electrode can be covered with a new compound adsorbed to its surface. In such case, we would detect its presence through the measured refractive index changes.

The desribed research emphasized integration of interferometry and electrochemistry in one setup, utilizing a boron-doped diamond film as a link between two subsystems. Such BDD element working as an electrode and reflective surface replaces costly high-quality mirrors used in many photonics applications. The significant cost savings on the optical part also stem from the need of only a light source, detector and optical fibers. The use of so few optical components and the lack of additional micromechanical elements with precise alignment requirements drastically reduces the cost of construction. The use of fibers in optical process control is possible due to their dielectric nature: they do not impact the electrochemical process. Further, as we analyzed the optical data in spectral domain, robustness with respect to possible optical transmission changes was achieved. Such an approach decreases the costs of the optical part and simplifies the construction, enabling the miniaturization. This may enable monitoring of electrochemical reactions in small valumes (e.g., a drop), reducing the sample amount needed to perform the experiment. On the other hand, performing separate measurements is time-consuming, requires more material for testing and people who can operate both systems. The proposed opto-electrochemical approach will simplify the measurement procedures and also reduce the amount of material needed to carry out the measurement. The possibilities of data analysis and insight into the interrogated material will be enhanced by examining possible correlations between the measured optical and EC parameters. Such hybrid NDE systems may prove particualry useful in examining electrode surfaces, in biomedical research, and in environmental analyzes (e.g., detecting the presence, and determining the level of a given substance in a liquid sample). As we offer the continuous measurements that utilize only one dual-role element, we open up a way to construct a lab-on-chip device.

## Summary and conclusion

We report a novel hybrid opto-electrochemical measurement system consisting of a 3-electrode electrochemical cell and a Fabry–Perot fiber-optic interferometer. The subsystems share a BDD film playing a dual role of a working electrode and a reflective layer. Simultaneous measurements were conducted during redox reactions in 2.5 mM K_3_[Fe(CN)_6_]/0.5 M Na_2_SO_4_ solutions. We obtained expected responses from both systems, confirming their proper NDE-mode operation without any sample alterations and no interference from one sub-setup to another. The simultaneous use of the BDD film as a reflecting layer in the interferometer and as a working electrode in an EC system has not been reported before. This novelty is advantageous in comparison to existing system architecture because of its simplified structure, reduced cost of construction, robust operation and system miniaturization possibilities for hybrid assessment of small droplet-sized sample volumes.

## Materials and methods

### Measurement setup

**Figure 5:**
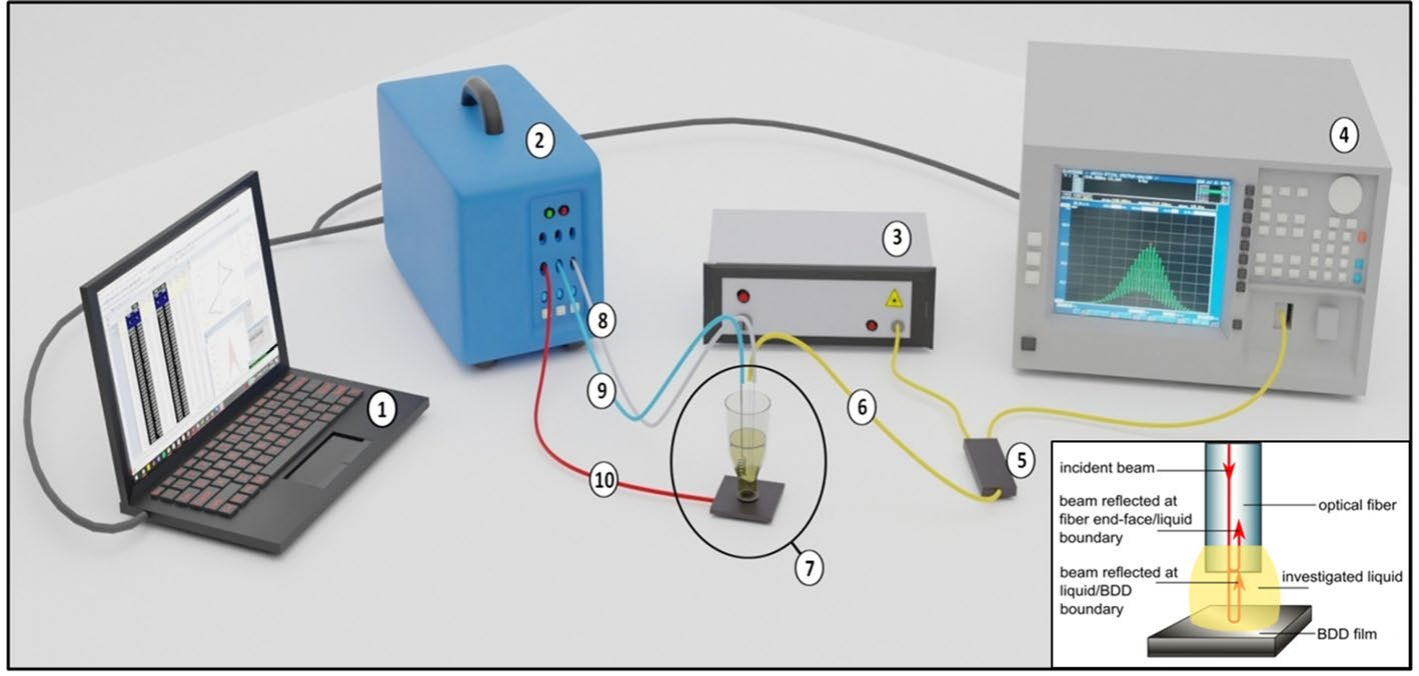
Measurement setup scheme (not to scale): 1—PC for storing and processing measurement data, 2—potentiostat–galvanostat, 3—light source, 4—optical spectrum analyser (OSA), 5—2 × 1 fiber coupler, 6—optical fiber, 7—measurement head, 8—connection to a reference electrode, 9—connection to a counter electrode, 10—connection to a working electrode. The inset figure shows a Fabry–Perot interferometer operation principle that was utilized in an optical part of the system. Reflected beams interfere with each other giving us a signal register by OSA.

The measurement setup presented in Fig. 5 consists of two main parts which have a boron-doped diamond film as a common element. Those two parts are a fiber-optic Fabry–Perot interferometer and a three-electrode electrochemical system.

Due to the water-based nature of the chemical solution, the optical measurements were performed with the use of the light source operating at 1550 nm. Hence, the Fabry–Perot part was build of a super luminescence diode working at a central wavelength of 1550 nm. For recording the optical spectra an optical spectrum analyzer was used. The single-mode standard communication optical fibers and a 2 × 1 fiber coupler were used to connect all the parts. The fiber that played the role of an optical measurement head was stripped and cleaved to fit the measurement field. The Fabry–Perot interferometer works in a reflective mode. Here, the incident beam is guided through the optical fiber and spitted onto a reference beam and a measurement beam. They are reflected from fiber end-face/medium inside the cavity interface and medium/BDD film interface, respectively. Changes in optical path difference affecting the phase difference between those two interfering beams depend on the refractive index of the medium and a geometrical path length. Their perturbations will modulate the measurement signal.

**Figure 6:**
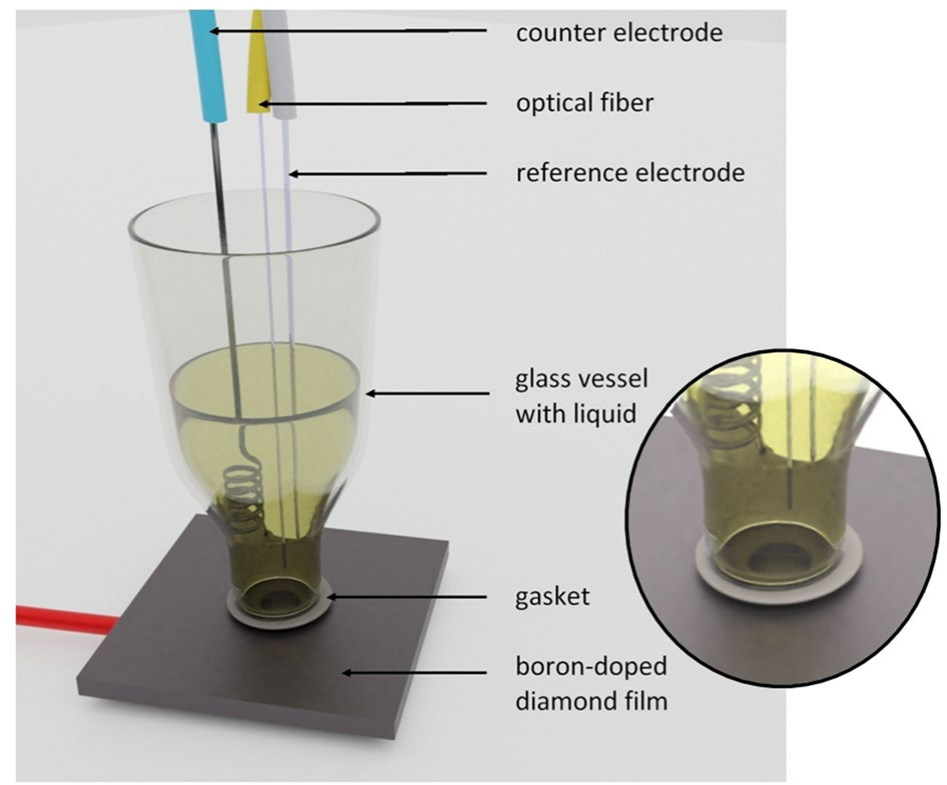
Measurement head scheme (not to scale). The counter, reference, and working electrodes are connected (blue, white, red wire, respectively) to the potentiostat–galvanostat, creating the electrochemical part of the measurement head. The optical fiber (yellow wire) is placed together with the electrodes in the glass vessel. The fiber and the boron-doped diamond film constitute the Fabry–Perot interferometer inside the electrochemical cell, with the cavity fulfilled by the investigated liquid. Close-up shows placement of the connections: all end above the BDD, they do not touch each other, and the optical fiber end-face is perpendicular to the BDD.

The electrochemical system was built in a standard three-electrode configuration: working electrode which was a boron-doped diamond deposited on a silica substrate, a silver wire with deposited silver chloride onto its surface as reference electrode and a counter electrode—Pt wire. The electrodes were connected to a potentiostat that controlled the potential and recorded voltammetric curves, which were observed on the PC. The close-up of the measurement head is shown in Fig. 6.

An electrochemical cell was placed over the boron-doped diamond with a fitted laser-cut gasket. The active area of the BDD electrode was 0.2 cm^2^. All the electrodes as well as the prepared optical fiber were immobilized on the laboratory stand and put inside the cell with special caution to assure their proper placement. It was crucial for the electrodes not to touch each other and for the fiber end-face to be placed parallel to the boron-doped diamond film.

### Chemicals

All reagents were of analytical grade and used without further purification. The Sodium sulfate (ACS reagent, purity ≥ 99%, Aldrich) and potassium hexacyanoferrate(III) (Pure p. a., Chempur) aqueous solutions were prepared using demineralised water. The Potassium nitrate (pure p.a.) and sulfuric acid (purity ≥ 95%) were obtained from Chempur. The synthesis gases: methane, hydrogen, and diborane were collected from Linde and were of the highest purity class.

### Boron-doped diamond film deposition

A 2.45 GHz microwave plasma-assisted chemical vapor deposition system (Seki Technotron AX5400S, Japan) was used for deposition of boron-doped diamond on 1 cm × 1 cm p-type silicon substrate with (100) orientation. All substrates were cleaned using the RCA method before deposition, followed by seeding using slurry consisting of undoped nanodiamonds with sizes ~ 4–7 nm. The reactor pressure, microwave power, gas flow rate were 50 Torr, 1300 W, 300 sccm, respectively. Diborane was used as a dopant precursor and the [B]/[C] ratio was maintained at 10,000 ppm. During the growth, the temperature of the graphite stage was kept at 700 °C. A deposition time was 12 h.

After the chemical vapor deposition process, the samples were cleaned in a solution of H_2_SO_4_ and KNO_3_ with a 2:1 weight ratio. First, the solution was heated until it reached its boiling point. Then the electrodes were placed in the boiling solution for 30 min and subsequently placed in the boiling deionized water. Next, the electrodes were ultra-sonicated in isopropyl alcohol and deionized water for 10 min. Finally, boron-doped diamond was hydrogenated in the previously mentioned system (1100 W, 50 Torr, 15 min).
